# Effects of advanced platelet‐rich fibrin combined with xenogenic bone on human periodontal ligament stem cells

**DOI:** 10.1002/cre2.563

**Published:** 2022-03-26

**Authors:** Meo Nguyen, Thuy Thu Nguyen, Ha Le Bao Tran, Dang Ngoc Tran, Lan Thi Quynh Ngo, Nam Cong‐Nhat Huynh

**Affiliations:** ^1^ Department of Periodontology, Faculty of Odonto‐Stomatology University of Medicine and Pharmacy at Ho Chi Minh City Ho Chi Minh City Vietnam; ^2^ Laboratory of Tissue Engineering and Biomedical Materials, Department of Physiology and Animal Biotechnology, Faculty of Biology‐Biotechnology, University of Science Vietnam National University Ho Chi Minh City Vietnam; ^3^ Faculty of Public Health University of Medicine and Pharmacy at Ho Chi Minh City Ho Chi Minh City Vietnam; ^4^ Department of Dental Basic Sciences, Faculty of Odonto‐Stomatology University of Medicine and Pharmacy at Ho Chi Minh City Ho Chi Minh City Vietnam

**Keywords:** advanced platelet‐rich fibrin, growth factors, human periodontal ligament stem cells, xenograft bone substitute material

## Abstract

**Objectives:**

In this study, we aimed to investigate the effects of a mixture of advanced platelet‐rich fibrin (A‐PRF) and xenogenic bone substitute material (XBSM) on the proliferation and migration of human periodontal ligament stem cells (hPDLSCs) based on the in vitro release of growth factors.

**Material and Methods:**

The concentrations of platelet‐derived growth factor‐AB (PDGF‐AB) and vascular endothelial growth factor (VEGF) released by the A‐PRF‐XBSM mixture were estimated using enzyme‐linked immunoassay for up to 7 d. The A‐PRF‐XBSM mixture exudate was incubated with hPDLSCs. At Days 1, 3, 5, and 7, cell proliferation and migration were investigated by cell counting and wound‐healing assays.

**Results:**

PDGD‐AB and VEGF were released from the A‐PRF‐XBSM mixture exudate for up to 7 days. hPDLSCs were cultured in media with various concentrations of the A‐PRF‐XBSM mixture exudate and exhibited their proliferation and migration ability. Furthermore, the factors released from the 100% A‐PRF‐XBSM mixture exudate had a substantial effect on cell migration, whereas those released from 4% and 20% A‐PRF‐XBSM mixture exudates stimulated hPDLSC proliferation.

**Conclusions:**

A‐PRF‐XBSM mixture continuously released growth factors over 7 days and enhanced hPDLSC proliferation and migration. Therefore, A‐PRF in combination with XBSM might provide potential advantages for periodontal tissue regeneration.

## 1 INTRODUCTION

Periodontitis is a multifactorial chronic infection caused by a myriad of bacterial species that interact with the host's tissues and cells, which causes the release of a series of cytokines, chemokines, and inflammatory mediators. These activities result in the destruction of the periodontal tissue structure, containing the gum, periodontal ligament, and alveolar bone (Dye, 2012). Periodontitis treatment aims to eliminate the inflammatory process, prevent disease progression, and regenerate lost periodontal tissue (Bosshardt & Sculean, 2009). Since the late 1980s, guided tissue regeneration (GTR) has been the most promising treatment because it creates space for cell migration and prevents the formation of the long junctional epithelium (Bunyaratavej & Wang, 2001). Previously, for GTR, only membranes were used to prevent epithelial adhesion on the root surface, but with a lack of conductivity and growth factor (GF) secreting cells (Stoecklin, 2013). Recently, human periodontal ligament stem cells (hPDLSCs) have been cultured from periodontal tissue and found to exhibit numerous prominent properties, such as the competence to differentiate into different types of cells in vitro like osteoblasts, fibroblasts, and cementoblasts (Silvério et al., 2010). In vitro, hPDLSCs play a role in the repair and regeneration of lost tissues in periodontitis. Therefore, they are often the priority choice for repairing and regenerating periodontal tissue in periodontitis treatment. However, the source and number of autogenous hPDLSCs are limited, thus preventing their clinical use in treatments for periodontal tissue regeneration. To overcome this drawback, GFs must be applied to stimulate the cells responsible for periodontal tissue regeneration.

Platelet‐rich fibrin (PRF) is a second‐generation biomaterial of concentrated platelets, first described by Dohan et al. (2006). PRF contains platelets, cytokines, white blood cells, and GFs (Choukroun et al., 2006). Several studies, using PRF alone or that in addition to other biomaterials, such as autologous bone grafts and allogeneic, xenogeneic, and bone substitute materials, demonstrated that this usually provides good clinical results in the treatment of intrabony periodontal defects (Lekovic et al., 2012). However, studies have not evaluated the actual effect on periodontal tissue regeneration in clinical settings. Many previous reports have demonstrated that PRF stimulates the proliferation, migration, and differentiation of various cell types in vitro, such as osteoblasts, gingival fibroblasts, and periodontal ligament stem cells (Chang & Zhao, 2011; Dohan Ehrenfest et al., 2009; He et al., 2009; Tsai et al., 2009). Lately, a PRF production protocol at lower speed centrifugation speed, called advanced‐PRF (A‐PRF), was developed. A‐PRF allows for the gradual release of GFs for up to a 10‐day period (Ghanaati et al., 2014; Fujioka‐Kobayashi et al., 2017). Additionally, A‐PRF is often mixed with varied biomaterials such as bone substitute materials. Hence, a comparison with the GFs released from various biomaterials following coating with A‐PRF is worthwhile. A‐PRF favors the release of a higher amount of GFs than classical PRF, which might directly alter tissue regeneration (Kobayashi et al., 2016). Although A‐PRF is universally used alone or as a transporter for a diversity of biomolecules in regenerative dentistry, its potential when used in combination with various biomaterials still requires more scientific evidence.

Previous clinical studies indicated that PRF combined with xenogenic bone substitute material (XBSM) might improve the probing depth, clinical attachment level gain, and defect filling when compared to those with XBSM or PRF alone (Lei et al., 2020; Lekovic et al., 2012; Sezgin et al., 2017). These improvements could be due to raised concentrations of various polypeptide GFs in the surgical sites. Polypeptide GFs might promote soft tissue healing by enhancing angiogenesis and matrix biosynthesis during the process of wound healing. However, little is known regarding the release of GFs since update database on the A‐PRF‐XBSM mixture and its effect on hPDLSC behaviors is still unclear. Therefore, the objectives of this study were to examine the GF release from different concentrations of A‐PRF‐XBSM mixture exudates and to investigate different in vitro responses of hPDLSC, including proliferation and migration to those A‐PRF‐XBSM concentrations.

## 2 MATERIALS AND METHODS

### 2.1 Culture of hPDLSCs

hPDLSCs were supplied by the Laboratory of Tissue Engineering and Biomedical Materials, University of Science, VNU‐HCM. These cells were demonstrated to express mesenchymal stem cell markers, such as CD44, CD73, and CD90. These stem cells contained the multipotential to differentiate into different kinds of cells, such as osteoblasts and adipocytes, in vitro (Tran Hle et al., 2014).  hPDLSCs were cultured in a complete medium containing Dulbecco's modified Eagle's medium/nutrient mixture F‐12 (DMEM/F12; Sigma‐Aldrich, MO, USA) complemented with 10% fetal bovine serum (FBS; Sigma‐Aldrich, MO, USA), 100 µg/ml streptomycin (Sigma‐Aldrich, MO, USA), and 100 IU/ml penicillin (Sigma‐Aldrich, MO, USA) at 37°C with 5% CO_2_ until 80% confluence. The complete medium was replaced every 2 days. The hPDLSCs at passage 4 were used in all the experiments.

### 2.2 Preparation of A‐PRF‐XBSM mixture exudate

Three peripheral blood samples were obtained from three healthy volunteers, non‐smokers, and non‐drinkers, aged 20–30 years old. This study was approved by the Ethical Committee of the University of Medicine and Pharmacy at Ho Chi Minh City (protocol number 503/DHYD‐HDDD). All participants provided signed informed consents before inclusion.

Blood samples were drawn into 10 ml A‐PRF glass‐coated tubes (A‐PRF tubes, Process for Nice France) without anticoagulant, and centrifuged at 1300 rpm for 14 min using a Duo Quattro Centrifuge (Process for PRF, Rotor angulation 41.3°, centrifugal force (time) 200*g*/14 min). The A‐PRF clots were concentrated in the middle of the centrifuge tube, separated, and gently compressed using an A‐PRF Box (PRF Process, France) to drain the A‐PRF membrane. Then, the A‐PRF membrane was cut into small fragments using sterilized scissors and mixed with XBSM powder (Bio‐Oss; Geislith Pharma AG, Wolhusen, Switzerland, granularity 0.25–1.0 mm) in an Eppendorf tube at a ratio of 1:1 (wt/wt).

The A‐PRF‐XBSM mixture was incubated with DMEM/F12 (0.2 g/ml, according to EN ISO 10993‐12:2009) at 37°C for 24 h with continuous agitation, and its exudate was collected and centrifuged at 3000 rpm for 5 min (Hettich, Hamburg, Germany) to discard red blood cells. The exudate obtained was defined as 100% solution. Three A‐PRF‐XBSM mixture exudate concentrations were used in this study, 100% A‐PRF‐XBSM, 20% A‐PRF‐XBSM, and 4% A‐PRF‐XBSM.

### 2.3 GF quantification in A‐PRF‐XBSM mixture exudate using enzyme‐linked immunoassay (ELISA)

The levels of GFs, PDGF‐AB, and VEGF, from the A‐PRF‐XBSM mixture exudate, were determined at 1 h, 6 h, 24 h, 3 days, 5 days, and 7 days by ELISA. All A‐PRF‐XBSM mixture exudate samples were collected and placed in an incubator (Stuart, UK) at 37°C wherein GFs were evenly released over time. At the indicated time, the medium was harvested and replaced with 5 ml of fresh DMEM/F12. In addition, the accumulation of GFs at the respected times was also evaluated. The concentrations of PDGF‐AB and VEGF were quantified using human ELISA kits (Sigma‐Aldrich, MO, USA) following the instructions of the manufacturer.

### 2.4 Effect of A‐PRF‐XBSM mixture exudate on hPDLSCs proliferation

To evaluate the effect of the A‐PRF‐XBSM mixture exudate on cell proliferation, a cell counting method with a hemocytometer was used. Here, 100%, 20%, and 4% A‐PRF‐XBSM were used as the experimental groups. Complete medium was used as the positive control.

hPDLSCs were seeded into a 96‐well plate (10^3^ cells/well; Nunc, Denmark) and cultured for 24 h in complete medium at 37°C with 5% CO_2_. After 24 h, the complete medium was substituted for the A‐PRF‐XBSM mixture exudate at different experimental concentrations. These cells were further cultured for 1, 3, 5, and 7 days. At each foreshowed time point, cells were extracted using 0.25% trypsin/EDTA (Sigma‐Aldrich, MO, USA) and dyed with trypan blue (Sigma‐Aldrich, MO, USA) for cell counting using a hemocytometer (Marienfeld, Germany).

### 2.5 Effect of A‐PRF‐XBSM mixture exudate on hPDLSC migration

To study the effect of A‐PRF‐XBSM mixture exudates on cell migration, a scratch wound‐healing assay mimics cell migration during wound healing in vitro. hPDLSCs were seeded into six‐well plates (3 × 10^5^ cells/well; Nunc, Denmark) and cultured until reaching 80% confluence. A scratch was introduced into the single‐layer on each dish using a pipette tip (100–1000 µl tip; Thermo Scientific, USA). Non‐adherent cells were extracted by washing once with 1× phosphate‐buffered saline (PBS) (Gibco, USA). The cells were then serum‐starved overnight. The A‐PRF‐XBSM mixture exudates were added to each well. Complete medium and DMEM/F12 without FBS were used as positive and negative controls, respectively. The images of cell migration into the scratch area at 0 and 24 h were taken using an Olympus CKX‐RCD microscope (Olympus, Japan) set up with a DP2‐BSW microscope camera and then analyzed using ImageJ 1.52a software (USA).

### 2.6 Statistical analysis

All data were expressed as the mean ± standard deviation and analyzed by Stata Software version 13. By using one‐way analysis of variance followed by a post‐hoc test performed the statistical analysis. *p* < .05 was considered significant.

## 3 RESULTS

### 3.1 GF release from A‐PRF‐XBSM over time

**Figure 1:**
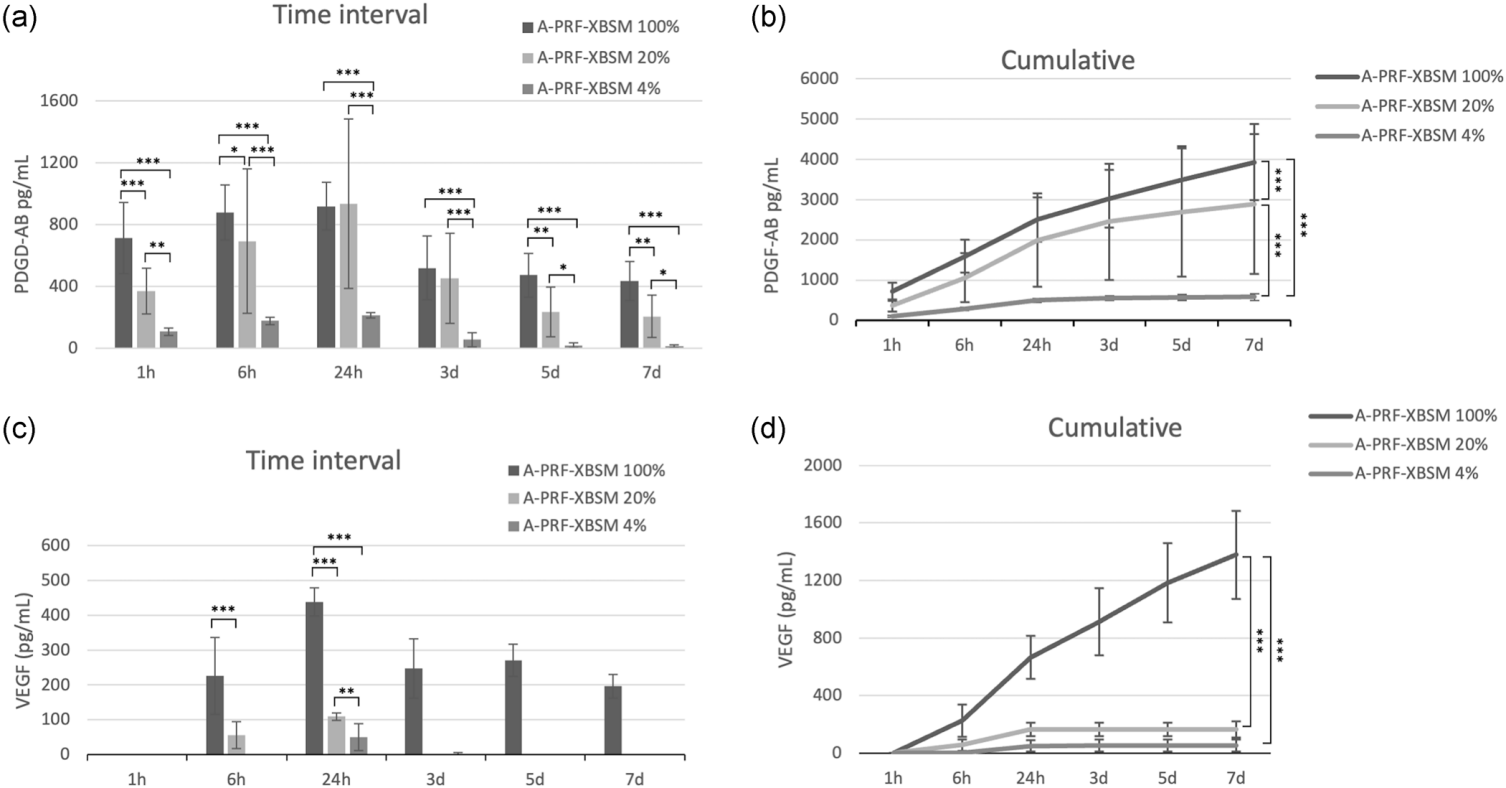
The amount at each time points of (a) PDGF‐AB, (c) VEGF over a 7‐day period. The total amount of GFs released over a 7‐day period for (b) PDGF‐AB, (d) VEGF. GF, growth factor; PDGF‐AB, platelet‐derived growth factor‐AB; VEGF, vascular endothelial growth factor. (*p* value: *p < .05, **p < .01, ***p < .001)

The release of GFs, including PDGF‐AB and VEGF, were analyzed at each time point, as well as at accumulation, as shown in Figure 1. After 6 h, PDGF‐AB and VEGF were released at significantly higher levels from 100% PRF‐XBSM than from 4% and 20% A‐PRF‐XBSM. The GF release trend from A‐PRF‐XBSM in various groups was increased in the early hours and peaked at 24 h, after which it gradually decreased at 7 days. In addition, total PDGF‐AB and VEGF accumulation was significantly higher in 100% A‐PRF‐XBSM than in 4% and 20% A‐PRF‐XBSM (*p* < .001).

A comparison between 4% and 20% A‐PRF‐XBSM demonstrated a significantly higher PDGF‐AB concentration in 20% A‐PRF‐XBSM than in 4% A‐PRF‐XBSM at all time intervals (Figure 1a). Correspondingly, the total accumulated PDGF‐AB and VEGF protein concentrations in 20% A‐PRF‐XBSM were significantly higher than those in 4% A‐PRF‐XBSM at Day 7 (Figure 1b,d). VEGF was released more slowly than PDGF‐AB based on the very small amount of VEGF after at 1 h in all groups. Furthermore, VEGF was maintained at a high level consistently more than 7 days with 100% A‐PRF‐XBSM, whereas the levels of VEGF from 4% and 20% A‐PRF‐XBSM were nearly undetectable from Days 3 to 7 (Figure 1c). The 100% A‐PRF‐XBSM modality released greater amounts of total PDGF‐AB and VEGF than all other samples, whereas 4% A‐PRF‐XBSM released the lowest amounts at all time intervals (Figure 1b,d).

### 3.2 A‐PRF‐XBSM mixture exudate promotes hPDLSC proliferation

hPDLSCs were exposed to A‐PRF‐XBSM mixture exudate at various concentrations for 1, 3, 5, and 7 days. The proliferation of hPDLSCs was evaluated by the cell counting method using a hemocytometer. On Day 1, the control and experiment groups showed significant differences (*p* < .001). In general, in different culture media, the number of hPDLSCs gradually elevated in every group during the evaluation time.

**Figure 2:**
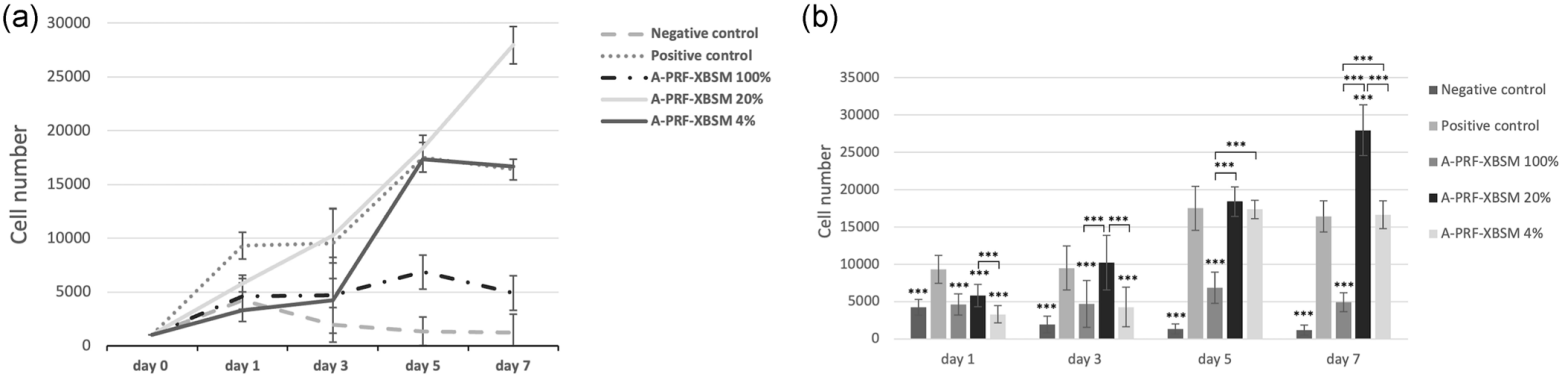
(a) The number of cells cultured in experimental media on Days 1, 3, 5, and 7. (b) Comparison effects of A‐PRF‐XBSM mixture exudate on the proliferation of hPDLSCs in various concentrations in vitro at different at time points. hPDLSC, human periodontal ligament stem cell. (*p* value: *p < .05, **p < .01, ***p < .001).

In the 4% and 20% A‐PRF‐XBSM groups, hPDLSCs had a similar tendency compared to proliferation in the positive control group; specifically, hPDLSCs numbers elevated from Day 0 to Day 1, and 3, peaked at Day 5 and decreased on Day 7 (Figure 2a). The results showed that 20% A‐PRF‐XBSM significantly promoted the proliferation of hPDLSCs compared to those treated with 4% and 100% A‐PRF‐XBSM at various time points (*p* < .001; Figure 2b).

### 3.3 A‐PRF‐XBSM mixture exudate promotes hPDLSC migration

**Figure 3:**
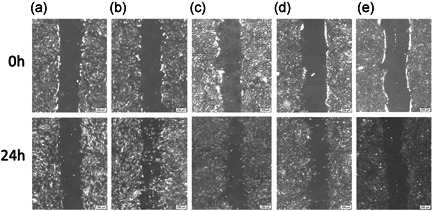
Migration of cell at 24 h after treatment (a) negative control; (b) positive control; (c) A‐PRF‐XBSM 100%; (d) A‐PRF‐XBSM 20%; (e) A‐PRF‐XBSM 4% (the percentage of hPDLSCs migration in experimental groups at 0 and 24 h). hPDLSC, human periodontal ligament stem cell

A scratch wound‐healing assay was performed to evaluate the migration of hPDLSCs incubated with different A‐PRF‐XBSM mixture concentrations. The groups incubated with A‐PRF‐XBSM displayed greater migration than the negative control group (Figure 3). In conclusion, hPDLSCs had greater migration competence when seeded in A‐PRF‐XBSM.

**Figure 4:**
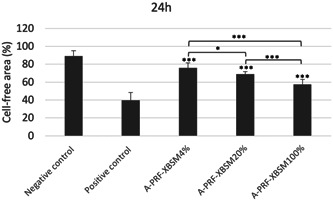
Comparison of the percentage of the cell‐free area among the experiment groups observed at 0, 24 h. (*p* value: *p < .05, ***p < .001)

Among the experimental groups, the migration capacity of hPDLSCs into cell‐free areas was observed after 24 h. Compared to that with the negative control, the percentage of cell‐free area for the positive control and 4%, 20%, and 100% A‐PRF‐XBSM groups decreased between 0 and 24 h (*p* < .001), indicating cell migration in these groups (Figure 4). A comparison of the groups showed significant differences in cell migration. The percentage of cell‐free in the 100% A‐PRF‐XBSM group was the lowest (57.71%). The migration capacity of hPDLSCs cultured in 100% A‐PRF‐XBSM was significantly higher than that in the 4% and 20% A‐PRF‐XBSM groups (*p* < .001). Additionally, the number of migrated cells in the 20% A‐PRF‐XBSM group was significantly higher than that in the 4% A‐PRF‐XBSM group (*p* < .05; Figure 4).

## 4 DISCUSSION

The biological gold standard for osseous regeneration is autologous bone based on its osteogenetic, osteoconductive, and osteoinductive properties. However, it undergoes rapid graft resorption and has some limitations. Hence, other bone substitute materials that are alternative to autologous bone are widely applied in dental fields. The most frequently used bone substitute materials are allogenic, xenograft, or alloplastic. Considering XBSM, mainly bovine origin, it has been demonstrated of osteoconductive property and a low absorbability rate (Schwartz et al., 2000). Therefore, to improve angiogenesis and osteogenesis ability, XBSM might be utilized in combination with autologous platelet concentrates to enhance successfully used in clinical.

Lately, the low‐speed centrifugation concept has shown a direct effect on the matrix content of PRF. Therefore, a new protocol for PRF processing that reduces the relative centrifugation force was recommended, which can yield a constant membrane in terms of A‐PRF. The primary difference between A‐PRF and conventional PRF is the decrease of centrifugation time and speed, which allows the maintenance of many important cellular components, such as the leukocyte number and the expression level of GFs are significantly increased in the PRF matrix were observed (Fujioka‐Kobayashi et al., 2016). Hence, in the present study, A‐PRF was chosen to examine its corporation with XBSM.

This study introduced an innovative method to combine bone graft particles in a bioactive structure, mixing XBSM with autologous biomaterial. Then, we evaluated the GF release from it and its effect on hPDLSC characteristics. The previous studies have elucidated GFs release from PRF alone and further experimented on its subsequent impacts on the various cell type's activity. PRF could promote the release of main GFs, such as VEGF, PDGF, TGF‐β, and FGF (Su et al., 2009). The release of GFs from PRF indicated that the membrane maintains a continuous and constant release of GFs more than 7 days (Dohan Ehrenfest et al., 2009). In this study, PDGF‐AB and VEGF were gradually released from the A‐PRF‐XBSM mixture exudate for up to 7 days. In addition, A‐PRF alone was found to release GFs over time, from its indicative platelet formulations and induced the slow release of GFs for up to 10 days (Choukroun et al., 2017; Kobayashi et al., 2016).

The A‐PRF‐XBSM mixture exudate was expected to produce a large number of GFs. The release of PDGF‐AB and VEGF were significantly higher with the 100% A‐PRF‐XBSM exudate than with the 4% and 20% A‐PRF‐XBSM exudates at all time points. VEGF from 4% and 20% A‐PRF‐XBSM was not detected after 24 h, whereas PDGF‐AB was continuously released until 7 days. This might be the products of the persistent platelets and remaining cellular units in the 4% and 20% A‐PRF‐XBSM samples. Moreover, the GFs continued to be released on Day 7 found in this present study. This result is analogous to other studies of PRF (Castro et al., 2019; Thanasrisuebwong et al., 2020).

As a periodontal tissue injury, alpha granules from platelets release a great amount of the PDGF‐AB and VEGF, which may act at the early stage during the healing period enhanced the cell mitogenesis, chemotaxis, and angiogenesis activities (Bennett & Schultz 1993). The results in this study indicated within the A‐PRF‐XBSM mixture exudate had the gradual release of PDGF‐AB and VEGF. It can demonstrate that A‐PRF incorporated with XBSM promotes the early healing process; this results agreed with the results of Castro et al. who investigated the concentrations of PDGF‐AB, VEGF from an L‐PRF membrane mixed with XBSM (Castro et al., 2019). Their study results indicated an analogous pattern of PDGF‐AB and VEGF release profiles from the PRF block. However, our study evaluated the discharge of PDGF‐AB and VEGF from A‐PRF‐XBSM, which also depends on the concentration of the A‐PRF‐XBSM mixture exudate.

An important evaluation was conducted in vitro regarding the effect of A‐PRF in a mixture with XBSM on hPDLSC characteristics. A‐PRF‐XBSM at various concentrations significantly stimulated hPDLSC migration and proliferation. Cell counting results demonstrated that 4% and 20% A‐PRF‐XBSM promoted the proliferation of hPDLSCs. However, 100% A‐PRF‐XBSM resulted during a decrease in proliferative activity, though it stayed higher than that within the control group. From this analysis, A‐PRF‐XBSM was able to stimulate the hPDLSC proliferation at an optimal concentration of 20%. As compared with literature, the varied concentrations of PRF were also analyzed for their effect on the proliferation ability of hPDLSCs. The effect of PRF is not only applied to different GFs but also to the three‐dimensional fibrin network structure of platelets, white blood cells, and GFs. However, the high A‐PRF‐XBSM concentration was unable to stimulate hPDLSC proliferation, which might be related to the pH value within the cellular environment. Previously, it has been demonstrated that the changes in platelet counts affected the pH value, which could negatively affect cell proliferation (Liu et al., 2002).

The wound‐healing assay revealed that A‐PRF‐XBSM promoted the migration of hPDLSCs at various concentrations. However, 4% and 20% A‐PRF‐XBSM resulted in a lower migration capacity than 100% A‐PRF‐XBSM. The high concentration of A‐PRF‐XBSM enhanced the migration of hPDLSCs; platelets and many different GFs bind to fibrin molecules, which facilitates cell components’ adhesion and creates a suitable microenvironment for the cell migration. In the study of Li et al., the effects of PRF exudate were evaluated based on the proliferation, osteogenic differentiation, and mineralization of hPDLCs in vitro, and the results showed that 100% PRF exudate could stimulate hPDLC proliferation and promote the differentiation of hPDLCs into mineralized tissue compare to 4% and 20% PRF exudate. These results are different from our study which could be explained by the fact that the exudate extracted from press PRF clots and conventional PRF was used in that study; whereas in our study A‐PRF was applied which demonstrated a larger amount GFs can have an impact on used concentrates (Li et al., 2018, 2019). This contributes to the main processes involved in periodontal tissue regeneration. The A‐PRF‐XBSM mixture exudate might provide potential benefits for periodontal tissue engineering and contribute to the dominant processes of regeneration (Kyyak et al., 2020; Thanasrisuebwong et al., 2020).

The management of intrabony defects involved different therapies, such as GTR, graft material, and biomaterial including platelet‐rich plasma, PRF, or a combination of the various methods (Kao et al., 2015). XBSM was used widely in regenerative dentistry, but it lacks osteoinductive capacity; as those combinations with bioactive agents as A‐PRF have a role biological connectors is to provide scaffolds with bioactive agents, cellular and signal molecules has been shown to stimulate the cellularity at the surgical site because of the continuous release of GFs and cytokines to be more effective in regenerating the alveolar bone (Avila‐Ortiz et al., 2016, Castro et al., 2019).

One limit of this study was that our study only estimated the release of PDGF‐AB and VEGF from A‐PRF‐XBSM mixture exudate; while there are a lot of GFs related to PRF that might function synergistically to promote wound healing and osteogenesis. Furthermore, we only assessed the proliferation and migration characteristics of hPDLSCs and GF release for up to 7 days, whereas the differentiation property of the hPDLSCs is one of the main keys for periodontal tissue regeneration, and GF release of platelet concentrates can continue until Days 14 or 28, as described in a previous study.

## 5 CONCLUSIONS

In this study, we found that A‐PRF combined with XBSM releases GFs, including PDGF‐AB and VEGF, for up to 7 days. A‐PRF‐XBSM further promoted the proliferation and migration of hPDLSCs in vitro; hence, A‐PRF‐XBSM might be effective for healing and periodontal regeneration.

## AUTHOR CONTRIBUTIONS

*Study conception and design*: Meo Nguyen, Thuy Thu Nguyen, Ha Le Bao Tran, Dang Ngoc Tran, Lan Thi Quynh Ngo, and Nam Cong‐Nhat Huynh. *Acquisition of data*: Meo Nguyen, Thuy Thu Nguyen, and Ha Le Bao Tran. *Analysis and interpretation of data*: Dang Ngoc Tran, Lan Thi Quynh Ngo, and Nam Cong‐Nhat Huynh. *Drafting of the manuscript*: Meo Nguyen, Thuy Thu Nguyen, Ha Le Bao Tran, and Nam Cong‐Nhat Huynh. *Critical revision*: Meo Nguyen, Thuy Thu Nguyen, Ha Le Bao Tran, Dang Ngoc Tran, Lan Thi Quynh Ngo, and Nam Cong‐Nhat Huynh.

## CONFLICTS OF INTEREST

The authors declare no conflicts of interest.

## Data Availability

The data that support the findings of this study are available from the corresponding author upon reasonable request.
